# Exploring the Landscape of Social Egg Freezing: Navigating Medical Advancements, Ethical Dilemmas, and Societal Impacts

**DOI:** 10.7759/cureus.47956

**Published:** 2023-10-30

**Authors:** Udit Lahoti, Sandhya Pajai, Tejas Shegekar, Anup Juganavar

**Affiliations:** 1 Obstretics and Gynecology, Jawaharlal Nehru Medical College, Datta Meghe Institute of Higher Education and Research, Wardha, IND; 2 Obstetrics and Gynecology, Jawaharlal Nehru Medical College, Datta Meghe Institute of Higher Education and Research, Wardha, IND; 3 Medicine, Jawaharlal Nehru Medical College, Datta Meghe Institute of Higher Education and Research, Wardha, IND

**Keywords:** cultural norms, egg freezing regulations, assisted reproductive technologies, reproductive autonomy, fertility preservation, social egg freezing

## Abstract

This narrative review article comprehensively explores the multifaceted landscape of social egg freezing, delving into its medical, ethical, societal, psychological, legal, and cultural dimensions. Oocyte cryopreservation, a developing procedure, gives women the chance to match their life goals with fertility goals. Informed decision-making, morally sound guidance, and open communication are all stressed by ethical considerations. Family planning practices, workplace cultures, and gender equality all have an impact on societal dynamics. The process's emotional toll and associated coping mechanisms are highlighted by psychological elements. Legal and policy frameworks need constant ethical reflection and understanding of the regulatory environment. Religious and cultural views highlight the variety of perspectives that influence attitudes toward this practice. For responsible practice to ensure individual liberty while navigating the evolving landscape of reproductive options, it is essential to comprehend how these aspects interact.

## Introduction and background

With the advent of social egg freezing, the field of reproductive medicine has seen a tremendous revolution recently. Egg freezing has evolved from being largely used for medical purposes, such as fertility preservation for those receiving cancer treatment, to being a proactive option for women looking to extend their reproductive possibilities past their peak childbearing years [1]. With this ground-breaking method, women may save their oocytes when their reproductive potential is at its highest and use them later in life when the conditions are more favorable for motherhood. When it comes to family planning, the idea of social egg freezing represents an important transition from the conventional timetables set by biological clocks and society norms [2]. Women are given the chance to have more control over their reproductive processes, which lessens time demands and enables them to make more informed life decisions. The emergence of this trend highlights the complex interactions between developments in medical technology, shifting societal perceptions, and changing ideas about motherhood and gender. 

It is crucial to carry out a comprehensive study of all of social egg freezing's complex aspects as it gains traction and popularity. This study seeks to go beyond the bare facts of medical practice and explore the moral, psychological, sociological, and economic ramifications of this revolutionary treatment. By breaking down each component, we hope to offer a comprehensive explanation of the phenomenon, illuminating both its potential advantages and the complex problems it poses. Addressing the mental health of the individuals involved requires an understanding of the factors that lead women to choose social egg freezing as well as the emotional effects of delayed parenthood [3]. To achieve a balance between personal preferences and social norms, it is important to thoroughly explore ethical issues related to autonomy, equality, and societal expectations [4]. Furthermore, it is important to consider the economic aspects because they are closely related to the decision-making process and affect how accessible this technique is [5]. In this review, our objective is to systematically examine the medical underpinnings, ethical quandaries, psychological impacts, and broader societal implications of social egg freezing. By doing this, we hope to contribute to a thorough dialogue that supports informed decision-making, enlightens policy discussions, and develops ethical practices in the always-changing field of reproductive medicine.

## Review

Methodology

**Figure 1:**
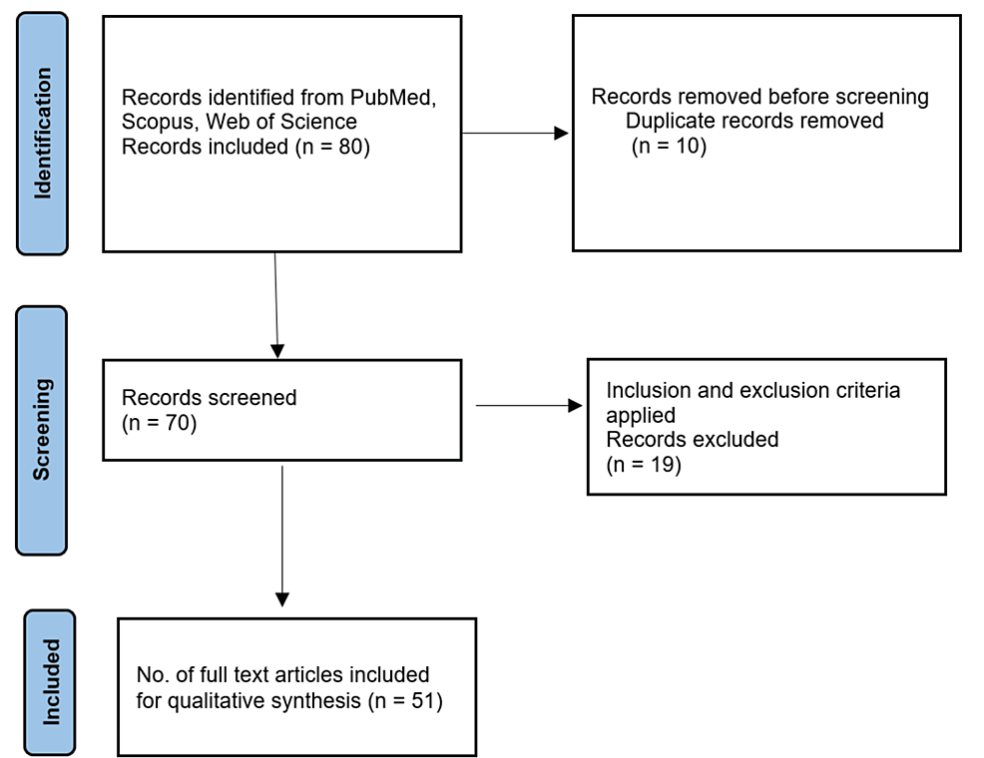
The selection process of articles used in this study.

In conducting this comprehensive review of social egg freezing, a search strategy was implemented to identify relevant literature across medical, ethical, and social dimensions. The search was carried out within several reputable electronic databases, including PubMed, Scopus, and Web of Science. To ensure the review's currency, publications from the year 2000 to September 2023 were considered, enabling the inclusion of the most recent developments and discussions in the field. A combination of keywords and controlled vocabulary terms, also known as MeSH terms, was utilized to enhance the precision and comprehensiveness of the search. These terms encompassed key concepts such as "social egg freezing," "fertility preservation," "oocyte cryopreservation," "ethical considerations," and "societal impact". In terms of inclusion and exclusion criteria, articles needed to meet specific requirements to be considered for this review. The inclusion criteria consisted of articles published in English that were directly related to social egg freezing, encompassing medical advancements, ethical dilemmas, societal impact, legal frameworks, and other relevant dimensions. Articles were limited to peer-reviewed publications, including original research, reviews, and scholarly articles. Non-English-language articles and those not directly pertinent to the scope of the review were excluded. The screening and selection process involved an initial evaluation of titles and abstracts to assess their relevance to the review's objectives. Subsequently, full-text articles were scrutinized based on the defined inclusion and exclusion criteria. Any discrepancies in the selection process were resolved through rigorous discussion among the authors, ensuring the integrity and consistency of the article selection process. In the final review, a total of 51 articles were deemed relevant and met the established criteria, collectively contributing to a comprehensive analysis of the multidimensional aspects of social egg freezing. To provide transparency in the selection process, a flow diagram that illustrates the article selection process will be included as an integral part of this review (Figure 1).

Medical dimensions of social egg freezing

Technological Advancements in Oocyte Cryopreservation

The practice of social egg freezing has undergone a revolution in the field of reproductive medicine with the introduction of sophisticated cryopreservation technology. The viability of thawed oocytes was impacted by traditional procedures utilizing slow-freezing technology because the formation of ice crystals frequently caused cellular damage [6]. However, the development of vitrification, a quick-freezing method that avoids the production of ice crystals, has significantly increased post-thaw survival rates [7]. Vitrification is the preferred approach in modern egg freezing practices because it has a better chance of maintaining oocyte quality and structural integrity [8]. Pre-freeze evaluation methods have also made improvements in the selection of viable oocytes for freezing. Now, it is possible to discriminate between oocytes with optimum and inadequate developmental potential using morphological and molecular criteria [9]. This improves not just the probability of oocyte survival post-thaw but also the likelihood of conceiving successfully after fertilization and implantation.

Assessing Fertility Preservation Options

For women considering postponing motherhood, social egg freezing is a feasible alternative alongside embryo freezing and ovarian tissue cryopreservation in the range of fertility preservation techniques [10]. Each approach has unique benefits and things to keep in mind. In order to develop embryos that may be frozen and kept, harvested oocytes must be fertilized with sperm. Women in committed partnerships who are also open to future in vitro fertilization procedures should pay particular attention to this strategy. Ovarian tissue cryopreservation, on the other hand, entails removing and freezing a piece of the ovary that contains primordial follicles with the goal of reimplanting the tissue in the future [11]. This approach is appropriate for people who may soon undergo fertility-threatening treatments like chemotherapy. The choice of preservation technique is made after taking into account each individual's medical history, marital situation, and long-term family planning objectives. Women must be able to weigh the benefits and drawbacks of each strategy in order to make judgments that are appropriate for their particular situation.

Medical Eligibility and Screening

The importance of ensuring applicants' appropriateness and safety increases as the practice of social egg freezing gains momentum. To determine if a person is qualified for the procedure and to uncover any underlying health issues that can compromise the effectiveness of the freezing and thawing process, a thorough medical assessment is crucial. Women undertake tests to ascertain their ovarian reserve, which represents the number and quality of their accessible eggs, before beginning social egg freezing. The follicle-stimulating hormone (FSH), anti-müllerian hormone (AMH), and antral follicle count (AFC) are three hormones that may be measured to determine the health of the ovaries [12]. These tests help doctors customize stimulation strategies for the best oocyte yield. A thorough medical history is taken to uncover any existing illnesses or prescription drugs that can influence the ovarian response to stimulation. Egg quality and quantity can be affected by conditions, including polycystic ovarian syndrome (PCOS), endometriosis, and certain drugs. Candidates are put through testing to rule out infectious disorders that may jeopardize the security of the freezing procedure for eggs. Human immunodeficiency virus, hepatitis B, and hepatitis C screens are frequently part of these exams [13].

Some facilities additionally conduct psychological tests to make sure that individuals are psychologically prepared for the procedure and its potential results due to the emotional and psychological effects of egg freezing [14]. Candidates get comprehensive explanations from doctors about the dangers and consequences of the egg freezing procedure. This gives women the ability to make well-informed decisions about whether to move forward and offers a chance to resolve any concerns. Clinicians create customized stimulation protocols to get the best egg production based on the findings of the ovarian reserve evaluation. Multiple follicles can be stimulated to grow and mature at the same time using drugs such as gonadotropins [15].

In summary, the medical eligibility and screening process makes sure that women who are thinking about social egg freezing are aware of the treatment, have reasonable expectations, and are in the optimum physical and psychological health to go through it successfully. This thorough approach aids in the procedure's overall success and safety.

Ethical contemplations

Autonomy and Decision-Making for Women

The practice of social egg freezing represents the idea of personal choice in reproduction. This ethical framework acknowledges the importance of personal agency and self-determination in choosing one's direction in life. Social egg freezing gives women the power to control their fertility by giving them the chance to save healthy eggs throughout their fertile years [2]. This proactive method gives women the freedom to break free from the limitations of biological deadlines by empowering them to coordinate family planning with their personal, educational, and career objectives. The ethical debate, however, also covers the factors that affect how women make decisions and goes beyond the act of storing eggs. The legitimacy of a woman's independent choices may be compromised by subtle influences from society, her family, and cultural norms [4]. True autonomy can only be attained by fostering a climate in which women are fully educated, unrestricted from outside influence, and allowed to make decisions that are consistent with their own aspirations [16].

Balancing Individual Choices and Societal Norms

Norms on when to have children were formed by social obstacles to egg freezing. There are worries about possible negative effects on maternal and child health despite the fact that it gives women the newfound flexibility to put off having children until they are better prepared to do so. This moral conflict serves as a reminder of the interdependence between personal goals and society's overall well-being [17]. The possibility of gender inequality being reinforced is a further ethical consideration. Social egg freezing may unintentionally promote the idea that women must choose between work growth and a family life by giving women a way to prioritize their career goals [18]. This emphasizes how crucial it is to overcome pervasive gender prejudices and promote a fairer distribution of caring duties.

It needs a comprehensive strategy that incorporates various viewpoints to navigate these moral dilemmas. It demands open discussions that take into account the objectives and issues of both sexes, as well as those of healthcare professionals, lawmakers, and the general public. To achieve a healthy balance between individual freedom and society’s well-being, it will be essential to ensure that reproductive decisions are based on well-informed judgment and that social institutions take shifting family dynamics into account.

Social dynamics and societal impact

Shifting Family Planning Norms

A fundamental change in conventional family planning standards has been brought about by the introduction of social egg freezing. Due to the availability of reproductive technology, traditional timeframes for establishing a family that were previously determined by biological elements are being rewritten [19]. By coordinating parenthood with their own personal, educational, and professional paths, this innovative method gives people more power to decide when to have children. Social egg freezing gives women the choice to prolong their reproductive window, testing the relationship between education, employment, and motherhood [20]. The way society views and accommodates the many life pathways of women might be altered as a result of this change. It also makes people rethink the expectations that society has for women to meet certain milestones. This change does, however, have ethical and cultural ramifications. Some see it as a chance for women to be liberated, while others express worries about the commodity of reproduction, the medicalization of aging, and the potential devaluation of the natural reproductive process. The cultural debate over these shifting family planning standards is a reflection of a larger discussion about personal freedom, decision-making, and the shared ideals that guide our conception of the family and motherhood [21].

Influence on Workplace Culture and Gender Equality

The practice of social egg freezing touches on issues of gender equality, workplace culture, and professional goals, adding to the continuing discussion about the challenges of juggling work and family responsibilities. The dynamics of the workplace are challenged to meet the changing requirements of their employees as more women experiment with delaying childbearing through egg freezing. One way that social egg freezing helps women is by removing the need to decide between advancing in their careers and having children at a physiologically ideal time [22]. It enables women to work towards their professional and educational objectives without giving up the chance to have children in the future. This may encourage a more inclusive and varied workforce, enabling women to succeed professionally while still pursuing their aspirations of becoming mothers. On the other side, the choice to postpone parenthood through egg freezing raises questions about the pervasive problems with gender equality in the workplace. Deeply rooted cultural prejudices may be seen in the idea that women must put off having children in order to prioritize their careers [23]. True gender equality necessitates the removal of structural barriers, the promotion of a supportive environment, and alternatives for delaying childbearing, in addition to offering opportunities for women and men to manage work and family obligations. In conclusion, the impact of social egg freezing on evolving family planning norms and workplace culture calls for a careful analysis of the structural changes and cultural values required to support actual gender equality.

Psychological and Emotional Considerations

The choice to pursue social egg freezing is a significant and complex one, characterized by a plethora of psychological and emotional issues. When considering this fertility preservation option, individuals and couples frequently find themselves negotiating a difficult terrain of emotions, concerns, and social pressures. In the process of social egg freezing, emotional stress is a significant factor. The doubt surrounding the procedure's success is at the heart of this tension. When deciding to use their frozen eggs in the future, many worry about the viability of the eggs and their possibilities of becoming pregnant in a healthy way. Anxiety and worry levels may increase as a result of this uncertainty [19]. The thought of postponing motherhood can also cause intense emotions, such as the worry that one will regret their decision. People may experience societal pressure to adhere to traditional family planning standards as a result of the expectation that they have children within a specific amount of time, which can further exacerbate mental stress [24].

Dealing with the psychological difficulties of social egg freezing is essential. To negotiate this emotional landscape, people frequently use a variety of coping mechanisms. A sense of connection and emotional comfort can be found by asking friends and family for social support. An important resource that helps people examine and deal with their emotional reactions in a planned and encouraging setting is psychological counseling or therapy. To deal with worry and stress, many people also use self-care techniques like journaling or mindfulness. According to research, people who receive counseling and emotional support are often better able to manage the emotional effects of reproductive decisions [25].

Social egg freezing has a psychological component that influences decision-making as well. It is not just a medical decision; it is also one that is highly emotional and intensely personal. Individuals may measure their own preferences against cultural standards and expectations, which may require thinking about their autonomy and sense of identity. As people attempt to find a balance between their objectives and the demands placed on family planning by society, ethical quandaries may occur [26]. For social egg freezing to be successful, psychological preparation is essential. It is crucial to tell them thoroughly about the emotional ramifications of the treatment and any potential psychological difficulties they could have. Giving people knowledge enables them to make wise decisions and better handle the subsequent emotional journey.

In summary, giving comprehensive and patient-centered treatment requires a grasp of and attention to the psychological and emotional aspects of social egg freezing. Understanding the emotional toll, giving coping mechanisms, respecting individuality, and offering emotional support are all factors that make the process of fertility preservation more kind and successful. Psychological and emotional considerations in social egg freezing are summarized in Table 1 [27-31].

**Table 1:** Psychological and emotional considerations in social egg freezing [27-31] The table has been created by the author themselves.

Psychological and emotional aspects	Impact of social egg freezing
Emotional strain	Coping with the decision-making process and uncertainty
Coping strategies	Seeking social support, counseling, and emotional outlets
Future expectations	Balancing expectations and managing potential disappointments
Decision-related stress	Navigating the emotional challenges of fertility choices
Personal identity and esteem	Impact on self-perception and identity
Relationship dynamics	Communicating with partners, family, and friends

Legal and policy frameworks

Regulatory Landscape and Future Considerations

In order to assure the appropriate and ethical use of these treatments, a careful examination of legal and legislative frameworks has been necessitated by the quick development of reproductive technologies, including social egg freezing [32]. Different nations and jurisdictions have different social egg freezing regulations, which address things like permission, storage time, information sharing, and insurance coverage. The legal framework governing social egg freezing is complex and constantly changing. Informed permission, storage restrictions, and the management of potential health hazards are only a few of the many topics covered in thorough guidelines several nations have devised to oversee the procedure's medical, ethical, and legal aspects [33]. There may be a complicated blending of clinical practices and ethical issues in other places due to a lack of particular regulation.

Future Considerations

Future concerns that need addressing as social egg freezing continues to grow in acceptance and significance include the need to give people thinking about social egg freezing clear, precise, and thorough information that should be emphasized in future laws. This makes sure that applicants are completely aware of the process, possible results, and related risks [34]. Policy talks may center on how long to keep frozen oocytes in storage, given the growing interest in expanding women's reproductive choices. It will be crucial to strike a balance between personal sovereignty and responsible storage techniques [32]. Concerns about social egg freezing's accessibility must be addressed by policymakers. For the sake of encouraging equitable reproductive options, it is crucial to make sure that this choice is accessible to a wide variety of people, regardless of socioeconomic status [35].

Maintaining patient confidence and preserving the procedure's integrity will depend on the creation and reinforcement of ethical standards for medical personnel, fertility clinics, and specialists involved in assisted reproduction [36]. The development of public education and awareness campaigns to educate people about the advantages, dangers, and potential drawbacks of social egg freezing may be the main emphasis of future policies [37]. Policymakers, ethicists, and healthcare workers must work together as the field of reproductive medicine continues to develop to make sure that legal and regulatory frameworks reflect the changing needs and values of individuals while preserving their wellbeing and autonomy.

Counseling and informed decision-making

Role of Counseling: Facilitating Informed Choices and Addressing Emotional Impact

Fertility counselors are crucial in providing complete information, guiding people through the complexities of the procedure, and helping them navigate the emotional complexities that come with deferred motherhood [38]. Counseling is crucial to the area of social egg freezing since it helps those who are contemplating it make decisions and maintain their emotional wellbeing. Fertility counselors offer a setting for making choices that are consistent with one's views and circumstances. Counselors explain the procedure, discuss potential results, and address any questions or concerns to help people understand the effects of social egg freezing and weigh the benefits and risks [39]. Choosing to freeze eggs can elicit a range of emotions, including excitement and hope, as well as anxiety and skepticism. In addition to offering techniques for coping with emotional stress and worry, fertility counselors also offer a safe space for clients to express their concerns [40]. The success of the process as a whole and the performance of the participants may be greatly influenced by this emotional support.

Fertility Education: Empowering Women With Comprehensive Information

Giving women comprehensive information regarding fertility is a crucial aspect of making well-informed decisions. Fertility consultants support this process by offering accurate information about the operation, the chance of success, potential risks, and long-term repercussions [41]. People are ensured, via this education, to have a thorough understanding of the possibilities available to them, enabling them to make decisions in keeping with their life goals.

Ethical Counseling Standards: Ensuring Ethical Practices in Fertility Centers

The duty of fertility counselors goes beyond simply dispensing knowledge; it also includes upholding moral principles that protect people's autonomy and well-being. In addition to ensuring that applicants are informed, ethical counseling guidelines also safeguard them against undue influence, pressure, or inflated expectations [42]. While guiding clients through the emotional and moral complexities of social egg freezing, counselors must uphold confidentiality, respect for others, and cultural sensitivity. Finally, counseling is a fundamental tenet of the social egg freezing method. It improves the entire experience and wellbeing of those starting out on this transforming journey by facilitating educated decision-making, addressing emotional difficulties, and upholding ethical norms.

Future prospects and ethical evolutions

Anticipating Social Shifts: Projecting the Future Role of Social Egg Freezing

As societal norms, technical developments, and ethical considerations continue to change, the function of social egg freezing is likely to see significant alterations. Social egg freezing may become more widely known and accepted as a legitimate method of delaying parenthood, changing the course of conventional family planning timelines [43]. Social egg freezing has the ability to decrease the reproductive opportunity gap between those who have more flexibility and those who do not, as it becomes more accessible and affordable [44]. Societies may see changes in employment policies, insurance coverage, and educational initiatives that support people's decisions to use social egg freezing [45].

Bioethical Considerations

Social egg freezing is one of many ethical, social, and philosophical issues that fall under the umbrella term of bioethical concerns. When it comes to social egg freezing for non-medical purposes, respecting human liberty and guaranteeing informed decision-making are fundamental bioethical concerns. It places a strong emphasis on the necessity of thorough instruction and guidance in order to empower people to make decisions in line with their beliefs and goals [26]. The questions of equality and access to social egg freezing are addressed by bioethics. A crucial ethical requirement is to guarantee that choices for fertility preservation are accessible to a wide variety of people, independent of their financial situation. Ethics experts support laws and procedures that advance egalitarian access [16]. The idea of reproductive freedom is frequently brought up in bioethical issues. It emphasizes the freedom of choice for people about their future reproductive health. In accordance with their own objectives, this includes the freedom to put off having children and to employ fertility preservation techniques [7].

Ethical Considerations

In the context of social egg freezing, ethical issues are particularly focused on the moral tenets and values that direct policy and practice. The use and regulation of fertility preservation technology are guided by these ethical principles. When considering social egg freezing, individuals must make educated decisions according to ethical considerations. This entails making sure that people are given accurate and fair information regarding the procedure's risks, advantages, and potential results. Allowing people to make decisions in line with their values and beliefs upholds the idea of autonomy [24]. The welfare and well-being of patients come first in ethical practices. This involves ensuring that social egg freezing medical procedures are performed in accordance with the highest standards of safety and effectiveness. Ethical rules encourage actions that put patients' best interests first [20]. The supervisory and regulatory structures are also subject to ethical issues. To avoid commercial exploitation, protect patient interests, and uphold the integrity of the fertility preservation process, ethical standards and laws are crucial. It is essential to have ethical governance to guarantee ethical behavior [19].

In conclusion, social egg freezing raises bioethical and ethical issues, highlighting the significance of patient welfare, autonomy, equity, and responsible governance. Collectively, these factors influence the moral foundation that underpins social egg freezing law and practice.

Long-term health and reproductive outcomes

Assessing Success Rates and Health Effects

People understandably worry about the long-term effects of social egg freezing on their health and ability to have children. Success rates and possible health impacts are two important factors to assess. Social egg freezing success rates take into account the likelihood of having a live baby using cryopreserved eggs. These rates are affected by a number of variables, including the woman's age at the time of egg freezing, the eggs' quality, the cryopreservation methods used, and the fertility clinic's level of experience [45]. To create reasonable expectations, people should have a realistic awareness of the success rates specific to their age group. Social egg freezing's implications for health are still being studied. Studies seek to ascertain if the act of egg freezing, or the subsequent usage of frozen eggs, has any effect on the mother's general health or the health of the children that are produced [46]. Long-term data are still few despite the fact that the majority of studies show no appreciable increase in health risks for women who undergo egg freezing. The success rates of social egg freezing and other fertility preservation techniques, such as embryo cryopreservation, may be compared to assist people in making educated selections based on their individual circumstances [22]. Success rates can range between treatments and should be taken into account along with other things, including future family planning objectives and medical background.

Regulatory agencies and professional associations are essential in making sure fertility clinics follow quality standards and correctly record success rates. It's crucial to comprehend the regulatory environment and the standards in place for reporting success rates to assess the accuracy of the data presented [47]. Understanding the possible short- and long-term health impacts of social egg freezing requires an open and honest discussion with healthcare professionals. Clinicians can give individualized advice based on a patient's medical background, health profile, and other pertinent information [48]. People should speak with experienced healthcare professionals, look for reliable information online, and weigh their personal beliefs and interests when making decisions about the long-term effects of social egg freezing on their health and ability to conceive.

Cultural and religious perspectives

Global Attitudes Toward Delayed Childbearing

Throughout the world, social egg freezing and, consequently, postponed childbirth are perceived from a variety of cultural and religious perspectives. There is a wide range of viewpoints on this practice since attitudes are influenced by societal conventions, values, and religious beliefs. The acceptance of delayed childbirth and reproductive technology is rising throughout many Western countries. Individual freedom and the pursuit of educational and professional objectives are frequently given priority. These principles are in line with social egg freezing, which gives women the choice to maintain their fertility while pursuing other goals in life [19]. Certain forms of conservative religion, including Catholicism and Orthodox Judaism, may have objections to reproductive procedures that require freezing eggs or embryos [49]. These viewpoints often prioritize natural conception and procreation within the context of traditional marriage.

Various viewpoints on reproductive technology exist within Islamic cultures. While the protection of life is typically emphasized in Islamic bioethics, believers and scholars may have different views on the morality of assisted reproductive technologies like egg freezing [27]. Family values and filial piety may have an impact on views towards childbirth in some Asian cultures. Perspectives on delayed childbirth and fertility preservation may be more complex if conventional expectations and contemporary options are balanced [50]. Other elements, including gender, social class, and geography, interact with cultural and religious viewpoints. People who have many identities must weigh complicated factors while deciding whether to engage in social egg freezing [51]. Exploring the complex interactions between cultural values, religious beliefs, and changing social norms is necessary to comprehend the views towards social egg freezing and delayed childbirth across the world. Healthcare providers ought to approach these talks with tact and knowledge of the various viewpoints that affect people's decisions. Cultural and religious perspectives on social egg freezing are summarized in Table 2.

**Table 2:** Cultural and religious perspectives on social egg freezing [19,37,48,50,51] Table was created by the authors themselves.

Cultural and religious perspectives	Attitudes toward social egg freezing
Western societies	Growing acceptance, aligning fertility with aspirations
Conservative religious views	Varying concerns about assisted reproductive methods
Islamic perspectives	Differing opinions based on interpretations
Asian cultural factors	Balancing familial values with modern opportunities
Societal norms and stigma	Pressure to conceive within specific age ranges
Intersectionality of identities	Complex considerations due to multiple identities

Summary of the studies included in the review is mentioned in Table 3.

**Table 3:** Summary table of studies included in the review

Study	Main Focus	Study Type	Key Findings
Petropanagos et al. (2015) [1]	Social egg freezing	Review	Ethical considerations and women's rights in social egg freezing.
Anbari et al. (2022) [3]	Fertility preservation strategies for cancerous women	Review	Reviews updated strategies for fertility preservation in women with cancer.
Shreffler et al. (2017) [5]	Responding to infertility	Review	Lessons from research and suggested guidelines for infertility practice.
Rienzi et al. (2012) [6]	Oocyte vitrification outcomes	Observational Cohort	Consistent and predictable delivery rates with oocyte vitrification.
Cobo et al. (2008) [7]	Fresh vs. cryopreserved donor oocytes	Comparative	Compares outcomes of fresh and cryopreserved donor oocytes.
Rienzi et al. (2009) [8]	Embryo development after ICSI	Randomized Controlled Study	Prospective study comparing development of embryos from fresh and vitrified oocytes.
Cobo and Diaz (2011) [9]	Clinical application of oocyte vitrification	Systematic Review and Meta-Analysis	Systematic review and meta-analysis of randomized controlled trials.
ASRM and SART (2013) [10]	Mature oocyte cryopreservation guidelines	Guideline	Provides guidelines for mature oocyte cryopreservation.
Donnez et al. (2015) [11]	Ovarian cortex transplantation	Commentary	Advocates for moving from experimental studies to clinical application.
La Marca and Sunkara (2014) [12]	Individualization of controlled ovarian stimulation	Review	Discusses individualized approaches using ovarian reserve markers.
Boitrelle et al. (2021) [13]	WHO manual for human semen analysis	Critical Review	Critical review and SWOT analysis of the sixth edition of the WHO manual.
Van den Broeck et al. (2013) [14]	Sperm donors: characteristics and attitudes	Systematic Review	Systematic review on demographic characteristics, attitudes, and motives of sperm donors.
Broer et al. (2014) [15]	Anti-Müllerian hormone and ovarian reserve	Review	Examines the clinical implications of anti-Müllerian hormone as a marker for ovarian reserve.
Browne et al. (2008) [16]	Ethical and psychological considerations	Commentary	Discusses ethical and psychological aspects in fertility preservation counseling.
Inhorn and Patrizio (2015) [19]	Infertility around the globe	Review	Examines global perspectives on gender, reproductive technologies, and infertility.
Borovecki et al. (2018) [20]	Social egg freezing under public health perspective	Ethical Case Analysis	Ethical case analysis of social egg freezing as a medical reality or women's right.
Tozzo (2021) [21]	Oocyte biobanks	Review	Discusses old assumptions and new challenges in the context of oocyte biobanks.
Hodes-Wertz et al. (2013) [22]	Women's perspectives on oocyte cryopreservation	Quantitative	Explores what reproductive-age women undergoing oocyte cryopreservation think about the process.
Pai et al. (2021) [24]	Oocyte cryopreservation: current scenario and future perspectives	Narrative Review	Provides a narrative review of the current scenario and future perspectives of oocyte cryopreservation.
de Boer et al. (2004) [25]	Number of retrieved oocytes in consecutive IVF cycles	Observational Cohort	Challenges the idea that the number of retrieved oocytes decreases in consecutive IVF cycles.
Dondorp and De Wert (2009) [26]	Fertility preservation for healthy women	Commentary	Examines ethical aspects of fertility preservation for healthy women.
Jadva et al. (2016) [28]	Characteristics and motivations of Indian egg donors	Quantitative	Explores characteristics, motivations, and feelings of Indian egg donors.
Sternke (2010) [31]	Ethical considerations of nonmedical preconception gender selection	Review	Examines ethical considerations in nonmedical preconception gender selection research.
Konc et al. (2014) [33]	Cryopreservation of embryos and oocytes	Review	Explores the cryopreservation of embryos and oocytes in human assisted reproduction.
Klitzman (2017) [34]	Ethical dilemmas among ART providers	Qualitative	Examines ethical dilemmas, views, and decisions among assisted reproductive technology providers.
Jackson (2018) [37]	Informed consent in social egg freezing	Review	Explores the ambiguities of 'social' egg freezing and challenges of informed consent.
Peterson et al. (2012) [38]	Infertility counseling	Review	An introduction to infertility counseling for mental health and medical professionals.
Letourneau et al. (2012) [39]	Pretreatment fertility counseling	Quantitative	Shows that fertility counseling and preservation improve quality of life in women with cancer.
Baldwin and Culley (2020) [40]	Women's experience of social egg freezing	Qualitative	Explores perceptions of success, risks, and 'going it alone' in social egg freezing.
Culley et al. (2013) [43]	Marginalization of men in infertility research	Review	Discusses the marginalization of men in social scientific research on infertility.
Jasienska (2020) [44]	Costs of reproduction and ageing in females	Review	Examines costs of reproduction and ageing in the human female.
Cobo et al. (2012) [45]	Outcomes of vitrified early cleavage-stage and blastocyst-stage embryos	Observational Cohort	Evaluates outcomes of vitrified early cleavage-stage and blastocyst-stage embryos.

## Conclusions

In essence, the conversation around social egg freezing has shown a complicated world involving technological advances, moral challenges, societal changes, psychological insights, legal frameworks, and cultural differences. This comprehensive knowledge is essential for encouraging ethical behaviors that uphold personal autonomy while taking larger issues into account. Women now have a way to match their reproductive goals with their life paths because of the advances in medical technology that have enabled oocyte cryopreservation. Medical issues, meanwhile, go beyond technological advances and also call for rigorous eligibility screenings, in-depth training, and well-informed decisions. The conflict between one's own autonomy and society’s expectations is highlighted by ethical reflections, underscoring the importance of ethical counseling, open communication, and the creation of guidelines that support patient wellbeing. The societal impact includes changing workplace cultures and family planning norms, rewriting the history of postponed motherhood, and promoting gender equality. Psychological aspects, however, emphasize emotional difficulties and resiliency in the face of this changing journey. To provide responsible and patient-centered care, the legal and policy frameworks need careful navigating of regulatory environments and ongoing ethical concerns. Regarding social egg freezing, cultural and religious perspectives emphasize how crucial it is to respect differing points of view. Fostering empathic healthcare relationships requires acknowledging the complex interplay of values and beliefs. In conclusion, a thorough comprehension of social egg freezing necessitates comprehending the interplay between technological advancement, moral standards, societal dynamics, psychological well-being, legal constraints, and cultural specificities. Responsible practices must sensitively negotiate this complex environment, giving people the information they need to make decisions that are in line with their beliefs and recognizing the constantly changing panorama of reproductive options.

## References

[1] Petropanagos A, Cattapan A, Baylis F, Leader A (2015). Social egg freezing: risk, benefits and other considerations. CMAJ.

[2] Inhorn MC, Birenbaum-Carmeli D (2008). Assisted reproductive technologies and culture change. Annu Rev Anthropol.

[3] Anbari F, Khalili MA, Mahaldashtian M, Ahmadi A, Palmerini MG (2022). Fertility preservation strategies for cancerous women: An updated review. Turk J Obstet Gynecol.

[4] Culley L, Hudson N, Rapport F, Blyth E, Norton W, Pacey AA (2011). Crossing borders for fertility treatment: motivations, destinations and outcomes of UK fertility travellers. Hum Reprod.

[5] Shreffler KM, Greil AL, McQuillan J (2017). Responding to infertility: lessons from a growing body of research and suggested guidelines for practice. Fam Relat.

[6] Rienzi L, Cobo A, Paffoni A (2012). Consistent and predictable delivery rates after oocyte vitrification: an observational longitudinal cohort multicentric study. Hum Reprod.

[7] Cobo A, Kuwayama M, Pérez S, Ruiz A, Pellicer A, Remohí J (2008). Comparison of concomitant outcome achieved with fresh and cryopreserved donor oocytes vitrified by the Cryotop method. Fertil Steril.

[8] Rienzi L, Romano S, Albricci L (2010). Embryo development of fresh 'versus' vitrified metaphase II oocytes after ICSI: a prospective randomized sibling-oocyte study. Hum Reprod.

[9] Cobo A, Diaz C (2011). Clinical application of oocyte vitrification: a systematic review and meta-analysis of randomized controlled trials. Fertil Steril.

[10] (2013). Mature oocyte cryopreservation: a guideline. Fertil Steril.

[11] Donnez J, Dolmans MM, Diaz C, Pellicer A (2015). Ovarian cortex transplantation: time to move on from experimental studies to open clinical application. Fertil Steril.

[12] La Marca A, Sunkara SK (2014). Individualization of controlled ovarian stimulation in IVF using ovarian reserve markers: from theory to practice. Hum Reprod Update.

[13] Boitrelle F, Shah R, Saleh R (2021). The sixth edition of the WHO manual for human semen analysis: a critical review and SWOT analysis. Life (Basel).

[14] Van den Broeck U, Vandermeeren M, Vanderschueren D, Enzlin P, Demyttenaere K, D'Hooghe T (2013). A systematic review of sperm donors: demographic characteristics, attitudes, motives and experiences of the process of sperm donation. Hum Reprod Update.

[15] Broer SL, Broekmans FJ, Laven JS, Fauser BC (2014). Anti-Müllerian hormone: ovarian reserve testing and its potential clinical implications. Hum Reprod Update.

[16] Browne H, Nurudeen S, Armstrong A, Decherney A (2008). Ethical and psychological considerations in fertility preservation counseling. Cancer J.

[17] Daniluk J (2001). Reconstructing their lives: a longitudinal, qualitative analysis of the transition to biological childlessness for infertile couples. J Couns Dev.

[18] Peterson BD, Pirritano M, Christensen U, Schmidt L (2008). The impact of partner coping in couples experiencing infertility. Hum Reprod.

[19] Inhorn MC, Patrizio P (2015). Infertility around the globe: new thinking on gender, reproductive technologies and global movements in the 21st century. Hum Reprod Update.

[20] Borovecki A, Tozzo P, Cerri N, Caenazzo L (2018). Social egg freezing under public health perspective: Just a medical reality or a women's right? An ethical case analysis. J Public Health Res.

[21] Tozzo P (2021). Oocyte biobanks: old assumptions and new challenges. BioTech (Basel).

[22] Hodes-Wertz B, Druckenmiller S, Smith M, Noyes N (2013). What do reproductive-age women who undergo oocyte cryopreservation think about the process as a means to preserve fertility?. Fertil Steril.

[23] Goldin C (2014). A grand gender convergence: its last chapter. Am Econ Rev.

[24] Pai HD, Baid R, Palshetkar NP, Pai A, Pai RD, Palshetkar R (2021). Oocyte cryopreservation - current scenario and future perspectives: a narrative review. J Hum Reprod Sci.

[25] de Boer EJ, Den Tonkelaar I, Burger CW, Looman CW, van Leeuwen FE, te Velde ER (2004). The number of retrieved oocytes does not decrease during consecutive gonadotrophin-stimulated IVF cycles. Hum Reprod.

[26] Dondorp WJ, De Wert GM (2009). Fertility preservation for healthy women: ethical aspects. Hum Reprod.

[27] Dyer SJ, Abrahams N, Hoffman M, van der Spuy ZM (2002). Infertility in South Africa: women's reproductive health knowledge and treatment-seeking behaviour for involuntary childlessness. Hum Reprod.

[28] Jadva V, Lamba N, Kadam K, Golombok S (2015). Indian egg donors' characteristics, motivations and feelings towards the recipient and resultant child. Reprod Biomed Soc Online.

[29] Lockwood GM (2011). Social egg freezing: the prospect of reproductive 'immortality' or a dangerous delusion?. Reprod Biomed Online.

[30] Pedro J, Brandão T, Schmidt L, Costa ME, Martins MV (2018). What do people know about fertility? A systematic review on fertility awareness and its associated factors. Ups J Med Sci.

[31] Sternke LM (2010). Ethical considerations of nonmedical preconception gender selection research. OJHE.

[32] Mertes H, Pennings G (2011). Social egg freezing: for better, not for worse. Reprod Biomed Online.

[33] Konc J, Kanyó K, Kriston R, Somoskői B, Cseh S (2014). Cryopreservation of embryos and oocytes in human assisted reproduction. Biomed Res Int.

[34] Klitzman R (2017). "Will they be good enough parents?": Ethical dilemmas, views, and decisions among assisted reproductive technology (ART) providers. AJOB Empir Bioeth.

[35] Pennings G (2013). Ethical aspects of social freezing. Gynecol Obstet Fertil.

[36] Cobb LN, Ke RW (2018). Ethical considerations in the field of assisted reproductive technology. Minerva Endocrinol.

[37] Jackson E (2018). The ambiguities of ‘social’ egg freezing and the challenges of informed consent. BioSocieties.

[38] Peterson B, Boivin J, Norré J, Smith C, Thorn P, Wischmann T (2012). An introduction to infertility counseling: a guide for mental health and medical professionals. J Assist Reprod Genet.

[39] Letourneau JM, Ebbel EE, Katz PP (2012). Pretreatment fertility counseling and fertility preservation improve quality of life in reproductive age women with cancer. Cancer.

[40] Baldwin K, Culley L (2020). Women's experience of social egg freezing: perceptions of success, risks, and 'going it alone'. Hum Fertil (Camb).

[41] Crespi C, Adams L, Gray TF, Azizoddin DR (2021). An integrative review of the role of nurses in fertility preservation for adolescents and young adults with cancer. Oncol Nurs Forum.

[42] Hashiloni-Dolev Y, Kaplan A, Shkedi-Rafid S (2011). The fertility myth: Israeli students' knowledge regarding age-related fertility decline and late pregnancies in an era of assisted reproduction technology. Hum Reprod.

[43] Culley L, Hudson N, Lohan M (2013). Where are all the men? The marginalization of men in social scientific research on infertility. Reprod Biomed Online.

[44] Jasienska G (2020). Costs of reproduction and ageing in the human female. Philos Trans R Soc Lond B Biol Sci.

[45] Cobo A, de los Santos MJ, Castellò D, Gámiz P, Campos P, Remohí J (2012). Outcomes of vitrified early cleavage-stage and blastocyst-stage embryos in a cryopreservation program: evaluation of 3,150 warming cycles. Fertil Steril.

[46] Haßdenteufel K, Müller M, Gutsfeld R (2023). Long-term effects of preeclampsia on maternal cardiovascular health and postpartum utilization of primary care: an observational claims data study. Arch Gynecol Obstet.

[47] Sauerbrun-Cutler MT, Reshef E, Has P, Frishman GN (2021). Society for Assisted Reproductive Technology advertising guidelines: How likely are member clinics to maintain compliance after resolving their violations?. F S Rep.

[48] Maheshwari A, Pandey S, Shetty A, Hamilton M, Bhattacharya S (2012). Obstetric and perinatal outcomes in singleton pregnancies resulting from the transfer of frozen thawed versus fresh embryos generated through in vitro fertilization treatment: a systematic review and meta-analysis. Fertil Steril.

[49] Verlinsky Y, Rechitsky S, Schoolcraft W, Strom C, Kuliev A (2001). Preimplantation diagnosis for Fanconi anemia combined with HLA matching. JAMA.

[50] Marcia C. Inhorn (2021). America’s arab refugees: vulnerability and health on the margins. J Refug Stud.

[51] Morgan LM, Roberts EF (2012). Reproductive governance in Latin America. Anthropol Med.

